# Evidence of within- and between-stock connectivity in Mediterranean fisheries challenges the stock unit paradigm

**DOI:** 10.1038/s41598-026-47826-7

**Published:** 2026-06-02

**Authors:** Georgios Kerametsidis, Manuel Hidalgo, Baptiste Mourre, Enrico Ser-Giacomi, Lucía López-López, Antonio Esteban, Encarnación García, Miguel Vivas, Angélique Jadaud, Grégoire Certain, Vincent Rossi

**Affiliations:** 1 Balearic Oceanographic Center, Group of Ecosystem Oceanography (GRECO), Spanish Institute of Oceanography (IEO, CSIC), Muelle de Poniente, s/n, 07015 Palma, Balearic Islands, Spain; 2 IMEDEA, Mediterranean Institute for Advanced Studies (CSIC-UIB), Esporles, Balearic Islands Spain; 3 Balearic Islands Coastal Observing and Forecasting System, SOCIB, Palma, Balearic Islands Spain; 4 IFISC, Institute for Cross-Disciplinary Physics and Complex Systems (CSIC-UIB), Palma, Balearic Islands, Spain; 5 Spanish Institute of Oceanography (IEO, CSIC), Oceanographic Center of Santander, Santander, Cantabria Spain; 6 Spanish Institute of Oceanography (IEO, CSIC), Oceanographic Center of San Pedro del Pinatar, Lo Pagán, Murcia, Spain; 7 MARBEC, Univ Montpellier, CNRS, Ifremer, IRD, Sète, France; 8 Mediterranean Institute of Oceanography, CNRS, Aix Marseille Univ, Univ. Toulon, IRD, Marseille, France

**Keywords:** Marine biology, Fisheries

## Abstract

Achieving sustainable management of marine resources and implementing biologically realistic assessment depend on accurate identification of stock structure. Most harvested marine species are currently assessed under the common and long-standing assumption of “stock unit” within designated spatiotemporally invariant polygons known as assessment units, despite evidence of passive and/or active dispersal across multiple scales. Our objective here is to investigate the connectivity and spatial structure of red mullet (*Mullus barbatus*) metapopulation system in the Northwestern Mediterranean Sea and compare the delineation of assessment units with biological units. To achieve this, we integrate simulated larval dispersal modelling with tissue stable isotope analysis, encompassing the entire life cycle. We observe misalignment between the present “closed” assessment units and the identified biologically informed units. The identified metapopulation system comprises northern and southern subpopulations with dissimilar demographic roles. This principally stems from the Northern Current mediated larval transport from high-density persistent spawning grounds and, secondarily, likely from the regional differences in the isotopic baseline associated with different productivity regimes. Such an interdisciplinary approach is of elevated importance for species with stark differences across their life history, such as red mullet. Moreover, larval connectivity diagnostics successfully capture the interannual variability of recruitment dynamics of this complex stock, evidencing the capacity of biophysical models to inform fisheries assessment and management. This study highlights the importance of implementing spatial stock assessment frameworks that rely on critical, yet still largely disregarded, ecological information.

**Supplementary Information:**

The online version contains supplementary material available at 10.1038/s41598-026-47826-7.

## Introduction

In ecology, dispersal refers to the movement of an individual or multiple individuals from their birthplace to the site where they reproduce, or the movement between successive reproductive sites^1^. Most organisms disperse actively and/or passively across one or more life stages, and aquatic environments provide especially diverse dispersal mechanisms. For certain benthic or sessile species, dispersal may be limited or maximized during the early life stages, primarily through larval dispersal, which refers to the movement from the spawning site to the settlement site^2^, while more mobile species may continue to disperse during juvenile and adult stages^3^. Dispersal plays a vital role in shaping biodiversity and species distributions, with significant implications for diverse processes such as resilience to climate change and increased fishing pressure, adaptation to habitat fragmentation, and the spread of invasive species^4–6^. Within the marine realm, dispersal over the early-life stages (henceforth ELS) is frequently associated with the passive advection during the egg and larval stages of many species that may not necessarily exhibit large-scale movements during the adult phase^7^. ELS dispersal increases the resilience and persistence of populations through replenishment and gene flow^8–10^. In some systems, well-studied and predictable dispersal pathways have given rise to a variety of dynamics with certain units classified as primarily “source” or “sink” (sub)populations, largely determined by the spatial complexity of marine populations^10–12^. Spatially complex populations present significant challenges in contemporary marine ecosystem management, particularly when they include keystone or commercially important species. Ignoring spatial structure has been shown to bias estimates of biological reference points^13^, potentially leading to mismanagement^14,15^. This effect can be even more pronounced for fish stocks that are overexploited, as is the case for many species in the Mediterranean Sea^16^.

Current stock assessment frameworks face numerous and varied challenges ranging from accurately estimating crucial life history parameters, selectivity, and ecological processes, among other factors, to accounting for all sources of uncertainty and properly fitting the data^17^. The lack of a unified nomenclature can also be perceived as an additional challenge. Although certain terms, such as ‘population’, are explicitly defined - for example, as a self-reproducing biological entity within which all individuals can interbreed and contribute to recruitment, often resulting in a distinct genetic unit^18^- others may be more ambiguous^19^. For instance, fishery “stock units” are often defined geographically rather than biologically^20^, with the term “stock” serving as a loose delineation often utilized to define management units, but which may constitute a population, metapopulation, sub-population, fishery unit, or a mixture of entities^18^. This discrepancy between the geographic and biological boundaries of stock units underlines the need to incorporate spatial dimensions into assessment frameworks^19,21,22^. There are three linked assumptions about the spatial component of stock unit in conventional stock assessment models: (1) the population is well-mixed, (2) closed, and (3) homogeneous, commonly referred to as “stock unit” assumption^19^. Individuals within a stock unit are also assumed to share identical vital rates, while fishing effort is presumed to be uniformly distributed^20^. However, growing evidence suggests that this assumption fails for many stocks, highlighting the need to incorporate spatial complexity into assessment models. While several spatially explicit stock assessment models have been developed^23,24^, their extensive data requirements limit their widespread application^20^. One of the greatest challenges in developing and implementing spatially explicit stock assessment models, especially when dealing with complex spatial structures, is first to obtain accurate connectivity data - such as movement rates and directions - and then to effectively integrate this information into the models^20^.

Marine connectivity is largely determined by oceanic circulation, particularly for organisms that are passively drifted during specific periods of their lifetime, making dispersal models essential for elucidating connectivity within and between fish stocks and for informing fisheries management. Stock structure has been associated with spatial patterns in phenotypic life history parameters, such as maturity, sex ratios, size, depth preference, recruitment etc.^25–27^. A suite of methods has been developed to define stock structure and quantify connectivity, with their suitability depending on the species examined, the geographic area, and the spatiotemporal scale of connectivity^28,29^. Some of the commonly used methods include molecular (e.g., genetics and omics), chemical (e.g., hard and soft tissue chemical composition), morphological (e.g., otolith morphometry) and artificial (e.g., tags) markers^30^ which over the years have provided a better understanding of the life history of many species, including some of the most emblematic such as European eel^31^ and Atlantic bluefin tuna^32^. Among these techniques, genetic connectivity derived from molecular markers has proven effective at large scales but is still controversial at fine spatiotemporal scales^33^, while tagging is also often inadequate for capturing connectivity processes that occur within narrow scales, especially for mobile species with life histories vastly different between the ELS and the adult phases. While otolith chemistry provides valuable insights into connectivity and stock unit structure, a growing body of research advises caution when using certain elements or isotopes for environmental life history reconstruction, as observed differences may be attributed to physiological rather than environmental processes^34,35^. For these reasons, ELS dispersal models are emerging as essential tools for quantifying the spatiotemporal variation in connectivity metrics^28,29^, which ultimately have a direct impact on the spatiotemporal variation of recruitment in fishery stocks. However, complementary approaches are also needed to provide connectivity information during the adult phase. Natural tags such as muscle tissue stable isotopes can reveal differences in trophic preferences, habitat use and, eventually, structure among population and subpopulations^36,37^. Tissue Stable Isotope Analysis (SIA) is a common technique in the field of trophic ecology and has been broadly utilized to reconstruct movements and migrations and inform on connectivity^36,38^, with carbon (*δ*^13^C) and nitrogen (*δ*^15^N) as the most common stable isotopes.

Improving fisheries management by understanding and accounting for the spatial structure and connectivity among populations may be particularly crucial for ecosystems with high levels of overexploitation. The Mediterranean Sea, a hotspot of marine biodiversity^39^, is recognized for its ecological and economic importance, hosting a plethora of commercially valuable fish species, most of which, including the red mullet in certain parts of the basin, are currently overexploited^16^. Accounting for spatial stock structure and understanding the connectivity among populations^21,39,40^ is a crucial element to improve the fisheries management in the Mediterranean Sea, yet conventional assessment methods often neglect the intricate ELS dynamics as well as complex spatial dynamics. ELS-mediated dispersal has already been investigated for some demersal species in the Mediterranean^41–44^. Among them, the red mullet *Mullus barbatus* is a commercially valued demersal species which is characterized by an early-life pelagic stage^45,46^. Various studies have attempted to investigate its population structure and connectivity using molecular and chemical markers^47,48^ across the whole basin, spatiotemporal models^49,50^ in the NW Mediterranean and dispersal simulations^51^ in the Central Mediterranean. However, to our knowledge, no prior study in the Mediterranean has leveraged the explanatory power of high-resolution particle tracking models with fine-scale spatiotemporal spawning site data. Moreover, this is the first time that modelled ELS dispersal is combined with muscle tissue stable isotopes that inform on possible trophic segregation during adulthood, with the aim of providing the most complete picture of connectivity for this bi-phasic species.

Here, by combining spatiotemporal information of known spawning areas, and potential settlement zones of two red mullet stock units in the NW Mediterranean, we apply a particle tracking model and white muscle tissue SIA to specifically assess (i) the mean pattern of between- and within-stock ELS connectivity applying prior spatiotemporal constraints and (ii) any potential between- or within-stock spatial segregation during the adult phase that informs on diet-driven population subunits. Finally, (iii) by comparing our metrics with demographic variables retrieved from official stock assessment reports, we place our results in the context of fisheries management and provide process-based insights suggesting the integration of these methods into spatial assessment models. Our results are discussed in the context of fisheries management and spatial stock assessment. In particular, we examine how ELS dispersal may challenge the current broadly used “stock unit” assessment framework in the Mediterranean Sea and beyond.

## Materials and methods

### Site and target-species description

**Fig. 1:**
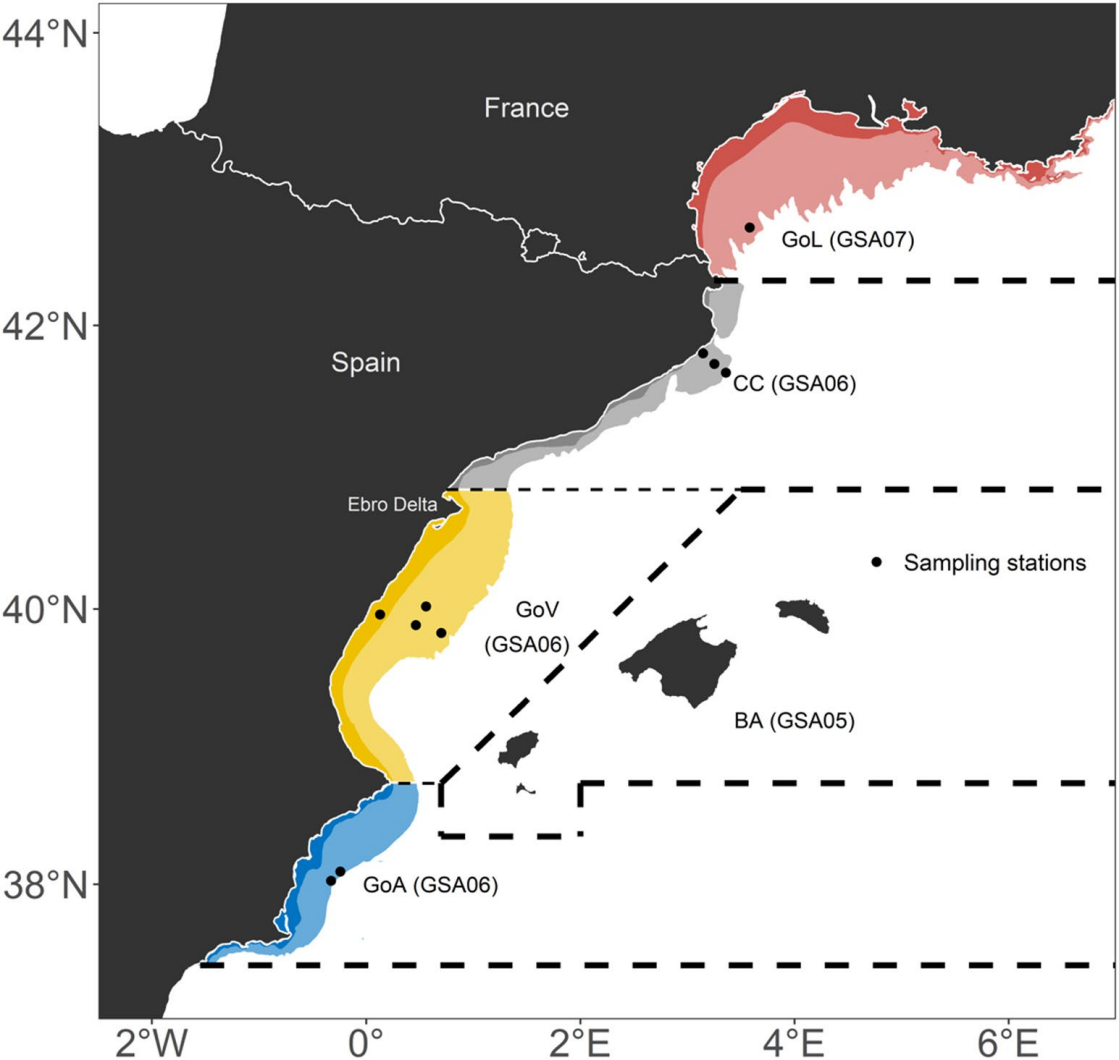
Map of the study area in the Northwestern (NW) Mediterranean Sea showing the sampling stations for white muscle tissue collection. Dashed lines indicate the boundaries of the subareas, while bold dashed lines indicate the boundaries of the current stock units (Geographic Subareas - GSAs). Dark shaded areas correspond to regions within the 50 m isobath (suitable settlement habitats), and light shaded areas represent regions within the 200 m isobath (potential spawning grounds). GSA07: Gulf of Lion (GoL) – in red; GSA06: Catalan Coast (CC) – in grey, Gulf of Valencia (GoV) – in yellow, and Gulf of Alicante (GoA) – in blue; GSA05: Balearic Archipelago (BA) – not coloured. The figure was created in R (Version 4.2.2, https://www.R-project.org).

The present study focuses on the NW Mediterranean Sea using the red mullet *Mullus barbatus* as a case study. This species ranks as the second and third most important demersal fish species in the area and the entire Mediterranean Sea, respectively, following hake and bogue^52^. Our study area encompasses two stock units recognized by the General Fisheries Commission for the Mediterranean and the Scientific, Technical, and Economic Committee for Fisheries: the Northern Spanish Coast (Geographic Sub-Area [GSA] 06) and the Gulf of Lion (GoL, GSA 07) in the NW Mediterranean. These regions serve as key assessment areas for red mullet (Fig. 1). The Balearic Archipelago (BA, GSA 05) was excluded from the analysis due to the species’ limited presence in the area. The BA’s seabed topography and sediment composition contribute to lower red mullet abundance, making it a less critical target for assessment.

The width of the continental shelf in the NW Mediterranean Sea exhibits considerable variation, stretching from a narrow corridor in the Northeastern GoL and along the Catalan Coast (CC) to expansive plateaus in the Western-Central Gulf of Lion, the Ebro Delta and Gulf of Valencia (GoV) (Fig. 1). One of the prominent features of the area is the Northern (or Liguro-Provençal) current, which flows southwest along the slope^53^. Upon reaching the Strait of Ibiza, a branch of this current diverts north-eastward, forming the Balearic Current^54^. Another branch of the Northern Current continues its south-westward flow along the Gulf of Alicante (GoA) until encountering the Cartagena-Tenés and Almeria-Oran fronts, which serve as significant oceanographic barriers. These fronts hinder gene flow^55^ and disrupt oceanic connectivity^56^ for many marine species. Henceforth, four distinct subareas in the NW Mediterranean Sea were identified based on their correspondence with spawning density hotspots^49,50^: the Gulf of Lion (GoL), which also constitutes a unique stock unit (GSA07), and the Catalan Coast (CC), Gulf of Valencia (GoV), and Gulf of Alicante (GoA) - collectively forming a separate stock unit (GSA06). Spawning density was derived from mature male and female abundance derived from observations from scientific trawl surveys.

In the Mediterranean Sea, red mullet attains commercial value by the end of its first year and is predominantly harvested by trawl and small-scale fisheries, with trawling accounting for the largest share of the catch^57^. The species is typically found on sandy and muddy bottoms at depths of up to 200 m and spawns from May to July^45,46^. Although it is a demersal species, its ELS is pelagic, making it susceptible to oceanographic advection with the pelagic phase lasting approximately 28–35 days before larvae settle on the seafloor^58^. Suitable settlement habitats are coastal regions with depths of up to 50 m, where small demersal individuals are abundant from late July to early September^46^.

### Biophysical modelling

#### Model description

To explore the ocean current mediated connectivity patterns of red mullet pelagic ELS, we employed the Lagrangian Flow Network (LFN) methodology^12,59–61^. LFN combines network theory tools and a particle-tracking model to simulate the transport and dispersal of fluid particles or any kind of passive tracers such as marine organisms ELS (i.e., eggs and larvae). It has been broadly applied to the study of marine connectivity^44,59,60,62^. A brief description is provided below, with additional details available in the relevant literature^12,59–61^.

As with most off-line particle tracking models, LFN can be coupled with any gridded two- or three-dimensional velocity fields from any source hydrodynamic model and, in turn, generate transport metrics corresponding to the input flow field. In the present study, LFN is coupled with high-resolution data-assimilative reanalysis simulations of the Western Mediterranean Sea for the period 2009–2019. The simulations were produced using the Western Mediterranean Operational modelling system^63,64^, developed at the Balearic Islands Coastal Observing and Forecasting System (www.socib.es). The model has a 2 km spatial resolution with 32 uneven terrain-following vertical layers providing a vertical resolution between 1 and 2 m at the surface. The model domain extends from Gibraltar Strait on the western side to the Sardinia Channel on the eastern side. It takes the outputs of the Copernicus Marine Service Mediterranean reanalysis^65^ as ocean boundary conditions and is forced at the surface with high-resolution atmospheric model outputs (5 km and 3-hourly before 2018 and 2.5 km and hourly from 2018) from the Spanish Meteorological Agency. Each year’s simulation is initialized on March 15th using ocean conditions from a 10-year-long free-run hindcast simulation which was shown to properly reproduce the mean circulation and the associated mesoscale activity^63,66^. The simulations integrate observations from satellite (sea surface temperature and sea level anomalies), in-situ platforms (Argo temperature and salinity profiles, fixed moorings) and the land-based HF radar in the Ibiza Channel. The local multi-model ensemble optimal interpolation scheme^67,68^ is used to assimilate the data with a 3-day assimilation cycle. The yearly simulations are run until 15 August, covering the whole spawning and larval drifting period of the species under study. The simulation outputs are saved every 6 h.

First, the LFN discretizes the WMOP domain (Western Mediterranean Sea) in a non-regular mesh of equal-area boxes, that correspond to the nodes of network and computes a land ratio for each of them (e.g., a node with a land ratio of 0/1 is considered fully covered by sea/land, respectively). The node size was set to a horizontal resolution of 1/12 degrees in a total N of 11,885 nodes. Then, to draw connections (i.e., network links) between nodes, we estimate water transport among each pair of nodes (boxes of the mesh). To this aim, taking a Lagrangian perspective, we fill each box with 100 ideal fluid particles^11^ and we track their trajectory in time. The tracking duration is adjusted to resemble the Pelagic Larval Duration (PLD), or the amount of time eggs and larvae spend in plankton. In our study, we did not account for any active larval behaviour while the PLD was set to 30 days based on available information from the literature^58,69^. Given that active larval movements during the greatest part of the pelagic phase should be minimal and swimming speeds extremely low^70^ as compared to the order of magnitude of horizontal currents (ranging 0.1 to 1 m.s^− 1^), this assumption is reasonable. Moreover, we chose to retain the lower end of the PLD range to mitigate any potential influence from minimal active behaviour that could occur in the later stages of the pelagic phase, when the individual may actively move towards a suitable settlement site. This is consistent with the large geographical focus and the mean scale at which connectivity metric is calculated (nodes with surfaces ranging approximately from 60 to 100 km^2^). Note also that it has been shown that simulated connectivity diagnostics for PLDs of 30 days are robust against uncertainties in the range of ± 7 days, minimizing any bias related to the retained PLD value^11^. The velocity field itself imposes the Courant-Friedrichs-Lewy condition, which is satisfied by a Runge-Kutta time step of 15 min. Just-released eggs and subsequent larvae are represented as passive particles drifting at a fixed depth of 10 m, in accordance with the present knowledge^71^.

Then, for every numerical experiment (corresponding to every starting date between the 1st of May and the 31st of July)^62^, a connectivity matrix is constructed recording each particle’s starting node *i* and ending node *j*. Such matrix includes all dispersal information and has as many rows and columns as there are nodes *N* in the network^60,62^.

The total number of particles originating from each subarea and successfully settling within the Western Mediterranean Sea, whether inside or outside the four NW Mediterranean subareas, was calculated. Additionally, the number of particles that settled specifically within any of the four NW Mediterranean subareas was also quantified.

#### Filtering

To restrict our analyses to only biologically relevant spawning (nodes where adults spawn and from which passive propagules can depart) and settlement (nodes where propagules can arrive and successfully settle) areas as well as to account for crucial life history traits of the species, a series of successive filters were superimposed on the connectivity matrices prior to computing the connectivity indices. The flexibility of LFN framework enabled us to add or remove any filter based on the degree of expertise or information available regarding the species under study and the degree of confidence to be given to each data component (e.g., Fig. S5-6). The filters we applied are as follows:

- ***Spawning quality filter***: The spawning quality of all potential sources (i.e.,nodes) was evaluated on the basis of the spawners density across the study area from Kerametsidis et al.^49^. More specifically, spatial standardised density indices (n/m^2^) were calculated annually based on fishery-independent survey data. We retained, averaged and normalized density indices over the study period (2009 to 2018) so that each node had a value ϵ [0,1] with higher values indicating high quality (Fig. S2). These values per node *i* were treated as factors and were multiplied by the connectivity matrices so that the number of transported particles from *i* to *j* nodes reflect not only current-driven dispersal pathways (of variable magnitudes) but also the spawning quality of *i*.
- ***Settlement suitability filter***: The suitability of a node as a settlement site was evaluated according to its bathymetry characteristics (see the dark shaded areas in Fig. 1). Nodes with a depth lower or equal to 50 m were considered suitable (= 1) for the settlement of red mullet ELS. Deeper nodes were considered unsuitable (= 0), so that all particles arriving there were considered lost.
- ***Temporal filter***: The spawning of the red mullet takes place between May and July. The 92 potential starting dates (1st of May till the 31st of July) were assigned a value $\:\in\:$ [0,1] with high values representing a great chance of spawning taking place in that date and vice versa. The 1st of June was considered the day with the greatest spawning potential with the rest of the days assigned a value that followed a normal distribution (µ = 0, σ^2^ = 20).

For each year from 2009 to 2018, the daily zero-inflated connectivity matrices indicating the inter-node particle transports as well as the daily connectivity indices (see below) have been reported online at 10.5281/zenodo.15916643.

#### Connectivity indices

Following Dubois et al.^12^, we estimated and analysed here four connectivity indices: local retention (LR), self-recruitment (SR), source-sink strength (SSS) and transport (T). These indices can be calculated per locality, where locality can be a single node or an agglomeration of nodes at sub-regional level (e.g., GoL). LR is defined as the ratio of locally produced settlement (i.e., arriving particles to a locality from which they were also produced) to local larval release (i.e., total number of departing particles from a locality) and SR as the ratio of locally produced settlement to the overall settlement^72^.

1 $$\:{LR}_{i}=\frac{Locally\:produced\:settlers\:in\:locality\:i}{Total\:locally\:released\:larvae\:from\:locality\:i}\:\in\:\left[\mathrm{0,1}\right]$$

2 $$\:{SR}_{i}=\frac{Locally\:produced\:settlers\:in\:locality\:i}{Total\:settlers\:in\:locality\:i}\:\in\:\left[\mathrm{0,1}\right]$$

For each locality, SSS was calculated as the number of particles arriving from other localities divided by the total number of particles exchanged, that is, the sum of particles arriving from other localities and particles departing locally. SSS values range from 0 to 1, where values close to 0 indicate source localities (net exporters of particles) and values close to 1 indicate sink localities (net receivers of particles).

3 $$\:{SSS}_{i}=\frac{Total\:arriving\:particles\:in\:locality\:i*}{Total\:arriving\:particles\:in\:locality\:i*\:+\:Total\:departing\:particles\:from\:locality\:i*\:}\:\in\:\left[\mathrm{0,1}\right]$$

where locally retained particles are excluded as * represents arriving (and departing) particles from localities other than *i* (i.e., ≠ *i*).

The Transport index was divided into two sub-indices. Transport of departure (T_d_) from locality A to locality B was defined as the ratio of particles departing from A and reaching B to the total number of larvae released in (A) Conversely, Transport of arrival (T_a_) from locality A to locality B was defined as the ratio of particles arriving in B from A to the total number of larvae released in (B). A full description of the indices can be found in Dubois et al.^12^.

Importantly, to assess the potential influence of the chosen prevailing depth of larval occurrence (10 m), we carried out a sensitivity analysis in which larval dispersal was simulated at 30 m. As in our main analyses reported here, in this alternative scenario, we calculated the number of particles imported to and exported from each area, as well as the inter-area larval transport (see Supplementary Information).

### White muscle tissue stable isotope analysis

Isotopes values are here expressed in delta notation (*δ*, parts per thousand deviations from a standard material), where ratios are expressed as: carbon *δ*^13^C = ^13^C:^12^C and nitrogen *δ*^15^N = ^15^N:^14^N. The isotopic composition was reported in the conventional delta (*δ*) per mil notation (‰), relative to Vienna Pee Dee Belemnite (*δ*^13^C) and atmospheric N_2_ (*δ*^15^N). Animal carbon *δ*^13^C and nitrogen *δ*^15^N stable isotope ratios mostly reflect the isotopic ratios of the animal’s diet^73^. However, beyond trophic position and dietary preferences, isotopic signatures have proven to be reliable indicators of species migrations^74^ and dispersal patterns, and geographical origins, reflecting their trophic environment^37^. This approach has been widely applied, particularly in estuarine^38^, but also in non-estuarine environments^36^. Variations in *δ*^15^N may reflect not only differences in the trophic level of prey species, but also variations in the baseline level of food webs^75^. Geographic variations in red mullet *δ*^13^C and *δ*^15^N values have been previously documented within a commercially important stock unit, the GSA07, and they were attributed to differences in prey composition and size^76^.

The sampling area included four subareas, in each of which, 15 individuals (whose mean lengths and standard deviations are provided in cm) were collected in late spring/early summer of 2021 (with the exception of just one single specimen collected in early autumn 2021) (Fig. 1, Table S2), as follows: GoL (16.82 ± 2.71 cm), CC (18.91 ± 2.16 cm), GoV (19.01 ± 1.57 cm), and GoA (18.52 ± 2.34 cm). Individuals were sampled from scientific and commercial operations: the majority of the samples originate from the EU-funded International Mediterranean Bottom Trawl Survey (MEDITS) that was carried out between spring and early summer (April–June) since 1994^77,78^. Only one specimen was collected during commercial fishing operations in 2021 (Table S2).

All the samples were frozen at -20 °C. Once in the laboratory for the analysis of the samples, a standard methodology was followed^79,80^. Firstly, muscle samples were thawed and dried for 48 h at 60 °C. Dry samples were then grounded to a fine powder using a hand mortar and pestle. The homogeneous dried powder (1–2 mg) of each biological sample was then placed into tin cups and then combusted by a continuous flow isotope ratio mass spectrometry (CF-IRMS) using a THERMO DELTA X-PLUS mass spectrometer in the University of the Balearic Islands. A standard protocol was followed, and a routinely used set of international reference materials was employed for the analysis of *δ*^13^C and *δ*^15^N. Finally, ANOVA, Kruskal-Wallis, t-test and Wilcoxon signed-rank tests were performed to test for any spatial differences in the isotopic data from different localities, depending on the number of groups and whether parametric test assumptions were met or not.

The collection and analysis of stable isotope samples was intended as a complementary and supportive analysis to the biophysical modelling, informing on the spatial structure of the meta-population at other life stage level. However, given the single-year collection and limited sample size, this analysis is used to test for patterns consistent with the modelled domains, not to definitively characterize long-term adult home ranges -which has previously been showcased for red mullet^49,50^- or diet.

### Links to stock assessment outputs

To explore potential relationships between fishery-dependent demographic data (extracted from the most recent stock assessment for red mullet; https://www.fao.org/gfcm/data/star/en/, accessed on March 2024) and our connectivity indices (Sect.  2.2), Spearman’s rank correlation coefficients were calculated between two sets of variables. Considering that such statistics do not theoretically ensure any causal links between both variables^81^, we report all tests performed (Fig. S7-8) but we only emphasize the significant correlations for which we could elaborate explanations that suggest causality. Three additional connectivity indicators were examined here: imports (i.e., the total number of arriving and successfully settled particles into a locality), exports (i.e., the total number of departing particles from a locality) and transport strength which represents the total number of transported particles. The former set includes recruitment (in thousands of individuals); spawning stock biomass *S* (in metric tonnes); recruitment success (calculated as the ratio of in the current year to the of the previous year); and residuals from the Ricker stock-recruitment relationship^82^. The Ricker model has already been successfully used for red mullet in the Mediterranean Sea^83,84^. Due to the relatively short lifespan of red mullet^85^, all individuals of age-2 or older are included in the spawning stock biomass. Consequently, correlations between demographic and connectivity indices were tested against *S* and residuals from the Ricker stock-recruitment relationship with a lag of one and two years, respectively. Conversely, *R* and *RS* correlations were only tested with a lag of zero and one years, respectively. To better account for the effect of connectivity indices on fishery-dependent demographic data, it is crucial to use temporal lags constrained by biological arguments depending on the selected variables^44^. Note that this study is, to our knowledge, the second one in the Mediterranean Sea to draw links between connectivity metrics derived from a numerical larval dispersal model and outputs of conventional stock assessments^44^.

## Results

### Between- and within-stock larval dispersal

**Fig. 2:**
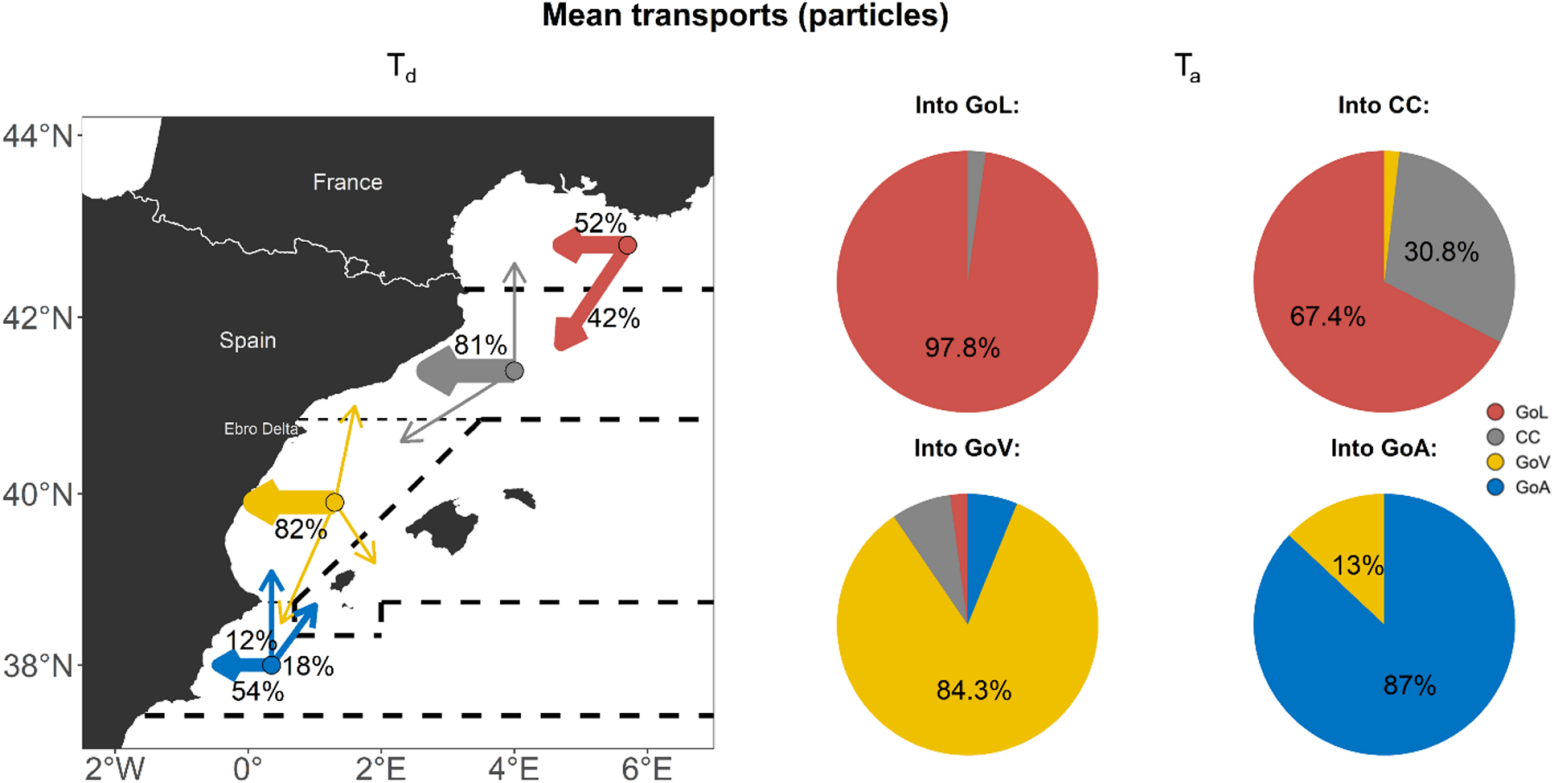
Mean transports of departing (T_d_) (map, left panel) and arriving (T_a_) (pie charts, right panel) particles. Percentages are based on the average total number of particles either departing from (map, left panel) or arriving into (pie charts, right panel) each subarea. Annotated arrows represent the outgoing transport flows (numbers indicate the magnitude), that is the departing/outgoing particles. Unannotated arrows represent outgoing transport flows ranging from 3% to 10%. Unlabelled segments in the pie charts represent incoming transport flows (namely, arriving/incoming particles) of 10% or less. The figure was created in R (Version 4.2.2, https://www.R-project.org).

We followed the trajectories of the released particles based on 92 annual spawning events between 2009 and 2018, totalling 920 spawning events (i.e., 92 spawning events per year * 10 years). Here, a spawning event corresponds to a spawning day that falls within the spawning period of the species (i.e., one of the 92 days between the 1st of May and the 31st of July). These data were used to quantify the transport of particles between pairs of subareas, with a focus on those either departing from or arriving at each subarea (Fig. 2).

Most particles originating from the GoL settled locally within the GoL itself (52%), with the second largest portion being deposited in the CC, accounting for 42%. In the CC, the majority of locally produced particles remained within the CC (81%), with minimal transport to the GoL and the GoV, each representing less than 10%. Similarly, 82% of particles produced in the GoV settled locally, with negligible transport to the CC, the GoA, or the BA. In contrast, nearly half of the particles originating from the GoA (54%) locally settled, while 18% were deposited in the BA and 12% in the GoV. Out of the total number of particles deposited from the GoL and CC, 5% ended up in suitable settlement sites (i.e., < 50 m deep) outside the examined five subareas of NW Mediterranean (e.g., the Alboran Sea or the Northern African Coast) (Fig. 2).

Nearly all particles that settled in the GoL originated from within the GoL (97.8%). In the CC, the majority of settled particles came from the GoL (67.4%), while 30.8% are produced and settled locally. In the GoV, most settled particles are locally produced (84.3%), with minor contributions from the neighbouring CC and GoA. Lastly, in the GoA, 87% of the settled particles were locally sourced, with the remaining 13% originating from the GoV (Fig. 2). Here, it is important to note that the percentages shown in Fig. 2 reflect not only dispersal dynamics but also the size of the examined subareas, which decreases southward across the study region.

Major transports occur between the two northern subareas, GoL and CC, and the two southern subareas, GoV and GoA, occasionally, highly variable (Fig. S3-4). In contrast, minimal or negligible transport is observed between the northern and southern subareas, possibly indicative of an ecological barrier separating the northern and southern regions of the NW Mediterranean which is located in the northern boundary of the Ebro Delta (Figs. 2 and 4).

### Connectivity indices

**Fig. 3:**
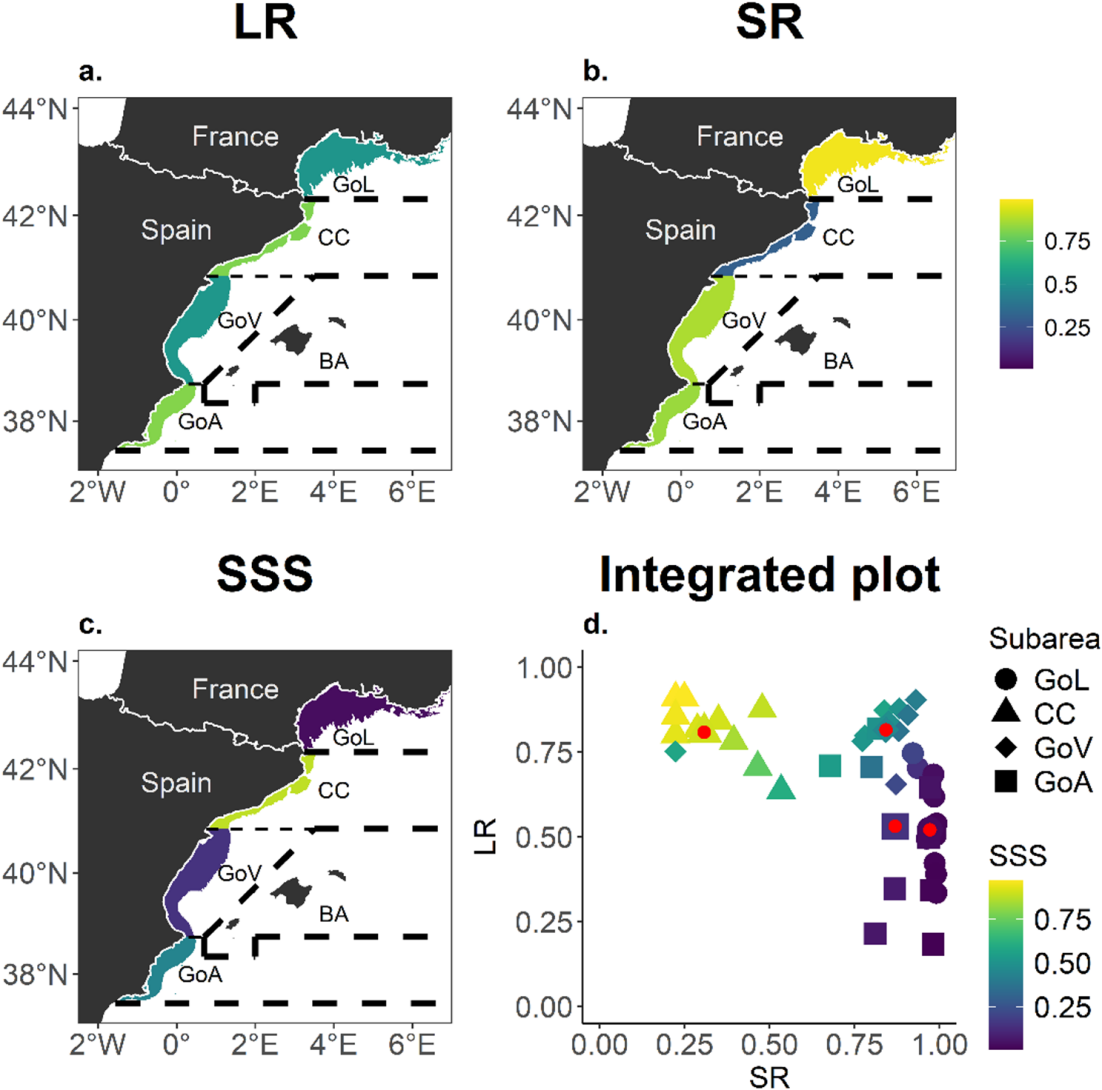
Average values of three connectivity indices: Local Retention (LR) (**a**), Self-Recruitment (SR) (**b**), Source-Sink Strength (SSS) (with values close to 0 representing sources and values close to 1 representing sinks) (**c**), and a combined plot integrating all three indices with each of the four point shapes representing a single year(**d**). Subareas are indicated as follows: GoL – Gulf of Lion; CC – Catalan Coast; GoV – Gulf of Valencia; GoA – Gulf of Alicante; BA: Balearic Archipelago. Red dotted shapes represent global average values. The figure was created in R (Version 4.2.2, https://www.R-project.org).

The GoL exhibited particularly high SR (0.97) and low SSS (0.03), with intermediate LR (0.52). In contrast, the CC displayed very high LR (0.81) and SSS (0.90) values, but low SR (0.31). The GoV displayed high LR (0.82) and SR (0.84), with an intermediate SSS (0.45). Lastly, the GoA exhibited intermediate LR (0.53), very high SR (0.87), and low SSS (0.15). Thus, the GoL is primarily a source of particles, while the CC serves mainly as a sink (Fig. 3, Fig. S1, Table 1).

**Table 1:** Average annual and global average (last row) values of three connectivity indices: Local Retention (LR), Self-Recruitment (SR), and Source-Sink Strength (SSS). Subareas are indicated as follows: GoL – Gulf of Lion; CC – Catalan Coast; GoV – Gulf of Valencia; GoA – Gulf of Alicante. Global averages refer to the average values across the ten years examined.

Year	GoL			CC			GoV			GoA		
	LR	SR	SSS	LR	SR	SSS	LR	SR	SSS	LR	SR	SSS
2009	0.54	0.99	0.01	0.82	0.29	0.92	0.81	0.88	0.37	0.71	0.68	0.54
2010	0.42	0.99	0.01	0.91	0.22	0.97	0.87	0.84	0.57	0.52	0.99	0.01
2011	0.70	0.94	0.14	0.71	0.46	0.73	0.88	0.88	0.49	0.50	0.97	0.03
2012	0.49	0.96	0.04	0.84	0.35	0.91	0.86	0.91	0.39	0.35	0.87	0.07
2013	0.62	0.98	0.03	0.78	0.39	0.85	0.66	0.87	0.22	0.21	0.81	0.06
2014	0.50	0.99	0.01	0.91	0.25	0.97	0.90	0.93	0.42	0.65	0.97	0.05
2015	0.39	0.99	0.01	0.80	0.22	0.93	0.80	0.78	0.53	0.82	0.82	0.50
2016	0.75	0.92	0.20	0.64	0.53	0.60	0.78	0.77	0.51	0.34	0.98	0.01
2017	0.68	0.98	0.04	0.88	0.48	0.89	0.84	0.85	0.48	0.18	0.98	0.00
2018	0.33	0.99	0.00	0.86	0.22	0.95	0.75	0.22	0.57	0.71	0.80	0.37
Average	0.52	0.97	0.03	0.81	0.31	0.90	0.82	0.84	0.45	0.53	0.87	0.14

### Muscle SIA

**Fig. 4:**
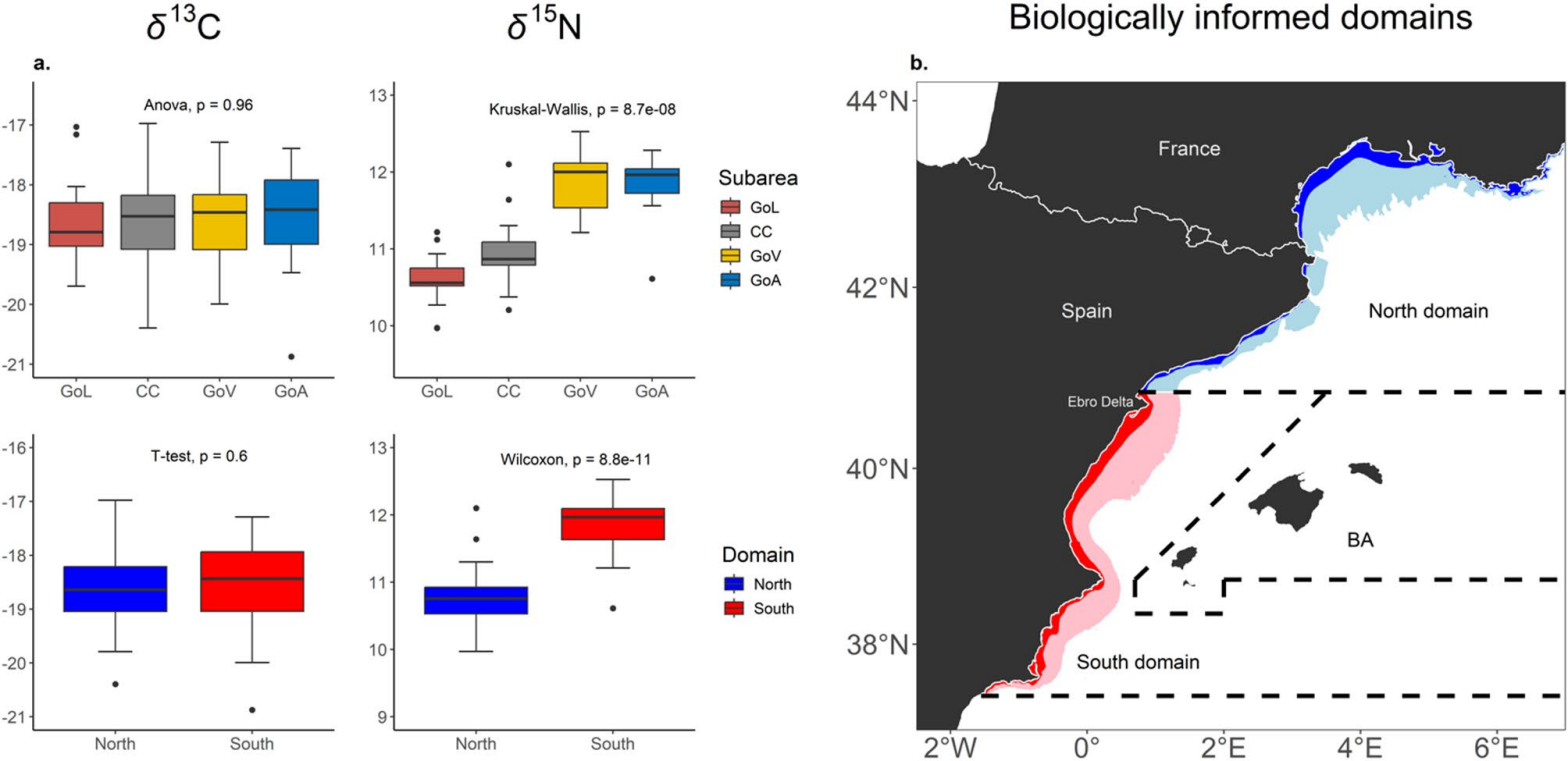
(**a**) Carbon (*δ*^13^C) and nitrogen (*δ*^15^N) isotopic ratios in the white muscle tissues of the collected specimens grouped in the four subareas (top left panel) or the 2 biologically informed domains that derived from ELS-mediated dispersal modelling (bottom left panel) and (**b**) the spatial domains corresponding to the biologically informed units. Dark shaded areas correspond to regions within the 50 m isobath (suitable settlement habitats), and light shaded areas represent regions within the 200 m isobath (potential spawning grounds). North domain – in blue; South domain – in red; GSA05: Balearic Archipelago (BA) – not coloured. The figure was created in R (Version 4.2.2, https://www.R-project.org).

Significant differences in *δ*¹⁵N were observed among the four subareas when analyzed collectively (*p* < 0.01). Additionally, when the subareas were grouped a priori into northern (GoL and CC) and southern (GoV and GoA) domains, significant differences in *δ*¹⁵N were also detected (*p* < 0.01). In contrast, no significant differences in *δ*¹³C were found either among the four subareas or between the northern and southern domains (*p* > 0.01) (Fig. 4a).

### Linking connectivity indices to recruitment (R), recruitment success (RS), spawning biomass (S) and the stock-recruitment (S-R) relationship

Connectivity metrics were related to several demographic variables that were retrieved from the official stock assessment reports for the GSA06 and GSA07 (Table 2). The following significant links were found between connectivity indices with the outputs of the stock assessment of the red mullet for the GSA06 (Fig. S7): *S* was negatively affected by LR of the GoV in June (*r*=-0.70, 1-yr lag) and the total LR of the GoA (*r*=-0.64, 2-yr lag); the residuals from the stock-recruitment relationship (S-R) were negatively linked to the T from the CC into the GoV during May (*r*=-0.75, 1-yr lag), the transport strength from the GoV to the GoA during May (*r*=-0.68, 1-yr lag) and the SR in the GoL during May (*r*=-0.72, 2-yr lag) and positively linked to the imports into the GoL in June (*r* = 0.76, 2-yr lag). No significant associations were found between connectivity indices and either recruitment or recruitment success (not shown).

**Table 2:** Overview of significant (*p* < 0.05) Spearman’s correlation tests between demographic and connectivity variables. Demographic variables include: Recruitment (R), Recruitment Success (RS), the residuals from Stock-Recruitment Relationship (S_R_resid) and Spawning Stock Biomass (S). Connectivity variables include: OUTWvec (exports): total of departing particles; INWvec (imports): total of incoming particles; T: Transport strength (number of transported particles); LR: Local Retention; SR: Self Recruitment; GSA07: Geographic Subarea 7 (Gulf of Lion) ; GSA06: Geographic Subarea 6 (Northern Mediterranean Spanish Coast); GoL – Gulf of Lion; CC – Catalan Coast; GoV – Gulf of Valencia; GoA – Gulf of Alicante. Demography and connectivity indices are calculated over the entire larval dispersal period (May–July), unless otherwise specified, to emphasize peak spawning months (May and/or June).

Demographic variable	Connectivity variable	Spearman’s *r*
RS_GSA07_lag1	T_CC_to_GoV_May	0.673
	SR_GoL	0.648
	LR_June_GoL	-0.636
S_R_resid_GSA07_lag1	OUTWvec_GSA07_May_June	0.709
	T_GoL_to_CC_May_June	0.709
	SR_GoL	0.697
	T_GoL_to_CC_June	0.697
	SR_June_CC	-0.685
	SR_GSA06_June	-0.673
	T_CC_to_GoV_June	0.673
	INWvec_GSA06	0.661
	INWvec_GSA06_May_June	0.661
	OUTWvec_GSA07_June	0.661
	LR_GSA07_May_June	-0.648
	OUTWvec_GSA07	0.648
	SR_GSA07_May_June	0.648
	T_GoL_to_CC	0.648
	INWvec_GSA06_June	0.636
	SR_June_GoL	0.636
	T_GoL_to_CC_May	0.636
S_R_resid_GSA07_lag2	OUTWvec_GSA06_June	0.648
S_R_resid_GSA06_lag1	T_CC_to_GoV_May	-0.745
	T_GoV_to_GoA_May	-0.675
S_R_resid_GSA06_lag2	INWvec_GSA07_June	0.758
	SR_May_GoL	-0.721
S_GSA06_lag1	LR_June_GoV	-0.697
S_GSA06_lag2	LR_GoA	-0.636

As for the GSA07 (Fig. S8), the recruitment success was significantly positively correlated with the transport strength from the CC into the GoV during May (*r* = 0.67, 1-yr lag) and the SR of the GoL (*r* = 0.65, 1-yr lag) and negatively correlated with the LR of the GoL in June (*r*=-0.63, 1-yr lag). Multiple variables were found to be positively and negatively associated to the residuals from the stock-recruitment relationship in 1-yr lag for the GSA07(see supplementary material). Lastly, the exports of the GSA06 in June were positively associated to the residuals from the stock-recruitment relationship for the GSA07 (*r* = 0.65, 2-yr lag). Connectivity indices showed no significant relationship with recruitment or spawning stock biomass (not shown).

## Discussion

Red mullet, a species of significant importance to Mediterranean fisheries, is currently assessed and managed as two distinct, presumably unconnected, panmictic, and self-sustaining stock units in the NW Mediterranean Sea, exploited predominantly by trawlers and small-scale fishing vessels from France and Spain^57^. To gain deeper insights into regional connectivity and stock structure throughout its biphasic life history, we combined the results of a particle tracking model and carbon and nitrogen stable isotope analysis focusing on the role of the main spawning grounds of the species in the NW Mediterranean. Our findings highlight substantial inter- and intra-unit larval connectivity in the NW Mediterranean and identify a critical species-specific boundary delineating two biologically informed spatial units pertaining to the same metapopulation structure: a northern and a southern domain, which is in direct contradiction with the current assessment framework based on GSAs 06 and 07.

The two biologically relevant spatial units identified here suggest that the Gulf of Lion serves as a vital and consistent source of larvae for the Catalan Coast, significantly influencing recruitment in the area. This implies that the current division between GSA06 and GSA07 (i.e., between Spain and France) strongly mismatches the biological structure of the species, which is consistent with scientific evidence for other species^44^. Further, the detection of a pronounced within-unit barrier in GSA06, which is likely to impact other species of commercial and conservation interest with similar larval dispersal characteristics and spawning phenology, challenges the functionality of the long-established spatial boundaries. Our findings demonstrate that significant within-unit spatial structure can emerge and persist, raising important questions about the adequacy of current assessment and management frameworks that ignore spatial and connectivity information. These results exemplify the impact of dispersal modelling on informing stock structure^86^, demonstrating the relevance of interdisciplinary approaches like ours. By combining dispersal modelling with muscle tissue SIA, we found that ELS-mediated dispersal patterns persist into adulthood, likely due to distinct isotopic baselines and limited large-scale adult movement. These results indicate two population units shaped by unique spawning hotspots, ocean circulation, and spatially distinct isotopic baselines.

In this study, by combining two methods targeting distinct and contrasting life stages of a highly commercial species, we first document intense connectivity during the pelagic ELS using dispersal simulations and, then, we demonstrate that these connectivity patterns are maintained into adulthood by analysing the stable isotopes of tissues of adult specimens from the NW Mediterranean. Previous interdisciplinary studies employing genetics, otolith chemistry and shape analyses, demography, parasites and life history parameters over the whole Mediterranean basin identified three or four different large-scale ‘megastocks’ of red mullet across the basin but failed to detect stock structure at finer scales^47,48^. Several authors have emphasized that the outcomes of genetic connectivity studies are highly sensitive to the spatial scales employed in the analysis^47,48^, with no clear consensus to-date^33,87^. Given the spatial extent of the study area and the extensive early life dispersal documented here, it is highly unlikely that genetic structuring exists within the NW Mediterranean red mullet metapopulation system^47,48^. While both particle tracking models and white muscle SIA have been used globally to study connectivity - either as standalone methods^29^ or in combination with others - this is the first study, to our knowledge, that attempts to fully integrate the results brought by two complementary techniques for this species. Our approach enables us to identify ELS-mediated transports while testing if the derived regional connectivity patterns are maintained through the demersal adult phase, itself characterized by the absence of large-scale movements^45,46^.

Particle-tracking models are widely used to infer marine connectivity pathways and have previously been applied to red mullet in the Strait of Sicily, Central Mediterranean Sea^51^. In the present study, each examined subarea, along with its prominent spawning grounds, experienced distinct oceanographic conditions, resulting in varying connectivity indices and transport patterns. Particular emphasis was placed on selecting spatiotemporal filters (representing spawning and recruitment) to more accurately simulate ELS dispersal^62^. Our findings indicate that the Gulf of Lion (GSA07) serves as a significant larval source, with nearly half of the locally produced larvae annually settling in the Catalan Coast, the northern region of GSA06. Additionally, the Gulf of Valencia and the Gulf of Alicante exhibit strong connectivity through ELS transport, and a persistent ecological barrier separating the North (Gulf of Lion and Catalan Coast) from the South (Gulf of Valencia and Gulf of Alicante) domains appears to persist into the adult phase, as evidenced by SIA. In contrast, in another system, significant larval exchange between stock units in the Strait of Sicily weas documented when only a particle-tracking model was used^51^. However, despite this exchange, unfavourable settlement habitats led to minimal successful settlement and, consequently, low effective connectivity - markedly differing from our results in the NW Mediterranean.

From a first order perspective, the Northern Current emerged as a major oceanographic feature driving larval drift in a predominantly south-westward direction, similar to what has been observed for other commercially important species like hake^44^ and gilthead seabream^41^. Somewhat contradicting this simplistic view, our results however clearly reveal a barrier just north of the Ebro Delta (~ 41°N), effectively dividing the NW Mediterranean red mullet metapopulation into two biologically distinct units. Several bio-physical factors can be invoked to support this latitudinal barrier. On one hand, the spawning phenology of our target species coincides with the seasonal slowdown of the Northern Current observed in late spring and summer, when its mean flow becomes shallower and its transport capacity decreases^88^, making cross-barrier transport highly unlikely for 30-day drifting durations despite the massive number of larvae produced in the Gulf of Lion. Regarding the larvae departing from the Catalan Coast, the sluggish Northern Current flowing far offshore^88^ combined with a narrower shelf that hosts only a few small high-quality spawning grounds (Fig. S2) as well as the intermittent wind-driven anticyclonic circulation north of the Ebro Delta^89^ favour the coastal retention of northern larvae (i.e., from the Gulf of Lion and the Catalan Coast) and limit their transport across the barrier. It should also be highlighted that the northern domain that we demonstrate here has also been suggested as a distinct hydrodynamical province for species with a PLD of 30 days^59^, identical to the one for the red mullet. The southern domain hosts a high spawning quality area - one of the most important red mullet spawning grounds in the NW Mediterranean^49,50^- off the Ebro Delta (Fig. S2) producing numerous larvae that are highly susceptible to remain confined there due to a weak, if any, south-westward drift and the formation of another wind-driven coastal anticyclonic eddy south of the Ebro Delta mouth^90^. Additionally, the mid-shelf spawning hotspot is spatially aligned with an area of low mesoscale activity and therefore prone to local retention, in contrast to the Catalan Sea that would be characterized by anisotropic transport^91^ with larvae drifting away from potentially favourable settlement areas.

The connectivity indices identified here were determined by a combination of hydrographical and biological factors, as well as the extent of each subarea. Due to the south-westward flow of the Northern Current, the presence of key spawning grounds in the Gulf of Lion^49^, and the region’s expansive size, this area emerges as a significant source of particles. It is characterized by near-zero particle inflow from the considered subareas, a very high self-recruitment, and the lowest local retention among the four examined subareas. However, it is important to acknowledge that, given the dominance of the Northern Current in the area^92^ and the presence of red mullet in the Ligurian Sea, a small percentage of ELS originating from the Ligurian region (not examined here) could successfully settle in the Gulf of Lion, affecting the connectivity indices in this subarea. However, considering the very narrow shelf in the eastern Gulf of Lion and the rare shelf intrusion of the Northern Current^93^, the likelihood of post-settlement survival of these incoming larvae should be relatively low. The Catalan Coast, due to its narrower continental shelf and the presence of important, yet highly dynamic spawning grounds^49^, receives a very high number of particles from the Gulf of Lion (also constituting the largest subarea in the system), which explains its low SR and SSS values (Fig. 3; Table 1). The Gulf of Valencia, constrained by the ecological barrier near the Ebro Delta, received only minor inflow from the north and, like the Gulf of Lion, was characterized by a high SR. In contrast, the Gulf of Alicante, the southernmost and smallest subarea in our study with a persistent high-density spawning ground^49^, exhibited low LR and SSS. This suggests that a small number of particles produced in the Gulf of Alicante -not exceeding 16% of the locally produced particles- may successfully settle outside the region (Figs. 2 and 3), potentially reaching areas in the southwestern Mediterranean such as the Algerian Coast (GSA04) or the Alboran Sea (GSA01). The high interannual variability of bidirectional early life stage transports among the Gulf of Valencia and the Gulf of Alicante is attributed to the high intra-^94,95^ or inter-annual^88^ variation of the transport of water masses in the Ibiza Channel.

In this study, our analyses were based on the best available biological data for the red mullet; however, it is essential to address certain potential limitations. We hypothesized that red mullet larvae are passively drifted for a period of 30 days. Various PLDs have been suggested in the literature ranging from 27 to 43 days for the Western Mediterranean Sea^58,69^, while another larval dispersal study in the Central Mediterranean used a PLD of 45 days^51^. Larval behaviour, including active vertical or horizontal movements, is a crucial factor to consider in dispersal simulations^96^. However, available information on red mullet larval behaviour is limited. Specifically, there is minimal data on vertical movement^71^ and no data on horizontal movements. Only slight diel vertical migrations of up to 10 to 20 m have been observed^71^. This relatively small variation, combined with speeds of several orders of magnitude higher in horizontal than in vertical current speeds^97^, confirms the outcomes of other studies in the Mediterranean that have documented that vertical migration of demersal species with a pelagic ELS phase might only exert minimal influence on dispersal patterns^98^. Moreover, our sensitivity analysis shows that depth has minimal effects on the connectivity metrics simulated here (Fig. S9-11), making our assumption on no-vertical migration reasonably justified. Specifically, imports and exports were generally found to be highly correlated between the two depth scenarios, while inter-area transport values typically differed by less than 10% (Fig. S9–S11). Regarding horizontal orientation, studies have emphasized its substantial influence on recruitment dynamics^99^. While active horizontal movements during the final stages of the pelagic phase may occur in red mullet larvae, further research on the matter is needed in this area. In our study, however, we chose a PLD of 30 days, excluding the potential impact of horizontal behaviours during the final pelagic stages. It is unlikely that larval swimming speeds (typically a few cm/s) would exceed the average surface speed of the Northern Current, the dominant factor driving the observed dispersal patterns. Similarly, circulation patterns near the Ebro Delta, where a barrier between two biological spatial units of red mullet is observed, indicate sufficiently high current speeds^90^ to influence any potential coastward active movements of larvae. Lastly, given the very low larval speeds and the large spatial extent of the four examined subareas -each spanning several thousand km^2^- it is highly unlikely that minimal horizontal movements would have significantly affected the transport patterns or connectivity indices calculated in this study over such broad scale.

Carbon and nitrogen stable isotope ratios are expected to reflect both the spatial variation in food sources and the trophic position of the studied species within the food web^73^. A critical factor influencing tissue isotopic composition is turnover rate -the time required for a tissue to regenerate and thus integrate the isotopic composition of the organism’s diet^100^. Turnover rates in fish can be highly variable, ranging from a few days to several months^101^, and thus isotopic values are commonly used for tracking animal movement. Hence, for non-migratory, resident species like the red mullet, isotopic signatures are expected to align with the baseline isotopic values of local food webs^38,75^. In this study, no significant intra- or inter-stock differences were observed in *δ*¹³C. As trophic enrichment in carbon isotopic ratios is small, variations in *δ*¹³C are commonly used to track the source of the trophic pathway and can be attributed to differences in algal or detrital carbon sources at lower trophic levels^102^ or may even reflect inputs of terrestrial organic matter^103^ or the balance between pelagic and benthic sources^79^. The lack of spatial differences in the carbon isotopic ratios of red mullet could stem from the homogeneity of the carbon isoscape in the study area, or the homogeneity in the diet of adult mullets. This species is known to have a diet rich in benthic invertebrates, including amphipods, decapods and polychaetes throughout the Mediterranean^76,104^, with evidence, in the study area, suggesting that the diet remains quite stable through its adulthood^105^. However, the spatial differences in *δ*¹⁵N observed in this study could partly reflect changes in diet and trophic level across space, with the species displaying a higher predatory role in the southern part of the study area. Moreover, spatial variation in *δ*¹⁵N associated with differences in diet has previously been documented in the Northwestern Mediterranean Sea^76^. The possibility of this variation stemming from dietary differences is highly unlikely, given that previous studies have identified a diet mainly based on polychaetes and suprabenthic crustaceans such as amphipods, shrimps and crabs along the Mediterranean coasts of Spain^105–107^, particularly for the size range of red mullet specimens used in this study. Thus, the large differences in *δ*¹⁵N suggest that the values could be mirroring the spatial patterns of nitrogen isoscape. This hypothesis is further supported by multi-species community data and the derived isoscapes for the NW Mediterranean (L. López-López, Pers. Comm., unpublished data), where the same pattern is generally observed over the whole community and used to estimate the spatial variation of the isotopic baseline^75^. Such a gradient reflected in the whole community of the region points toward differences in isotopic baselines rather than any other plausible scenario (i.e., dietary differences). Different isotopic baselines north and south of the Ebro Delta are consistent with two contrasting trophic pathways and biological productivity regimes, that also affect the regional distribution and dynamics of other species^108^. Regarding these findings, it is important to acknowledge that our samples were collected during a relatively short period, from summer to early autumn of 2021, and reflect the dietary intake of adult red mullet in the weeks or months prior to capture. To determine whether the identified spatial patterns are robust to potential interannual variations, implementing a consistent, spatially uniform (e.g., targeting the inner-, mid- and outer-shelf similarly in all GSAs) sampling effort across multiple years would be ideal. Additionally, the collection and stable isotope analysis of baseline, prey, and anchor species (i.e., widely distributed organisms of low trophic level) samples could help to further ascertain our interpretations and is recommended for future research.

Accurately describing spatial demographic variations, especially in growth, is crucial for fisheries assessment and management^17^. While most stock assessments still assume a panmictic population, our results suggest that ignoring underlying spatial dynamics may hinder efforts to achieve sustainable management goals^20^. Furthermore, ignoring biocomplexity (i.e., complex population or genetic structure within a species) may result in catastrophic consequences^109^, such as stock collapses^14^. Here, we demonstrated that stock-recruitment dynamics in either of the current assessment units are linked to several connectivity indices, indicating that connectivity processes play an important role in shaping recruitment dynamics. A striking example is the transport of particles between the Gulf of Lion and the Catalan Coast -two areas currently treated as separate stock units- highlighting the overlooked yet significant role of spatial stock structure. To our knowledge, this is one of the few studies in which a direct link between connectivity metrics and stock assessment outputs has been documented^44^. Beyond these correlations, recruitment success and residuals from the stock recruitment relationships are considered more representative indicators of the density-independent variability, which is often attributed to the complexity of environmental influences^110,111^. Our results suggest that connectivity and retention processes are included in these terms. Although multiple causal links (i.e., complex environmental processes) could underlie these significant relationships, connectivity metrics explain a large proportion of the variability⁴⁴. This highlights the importance of incorporating movement rates into assessment models, even for species whose dispersal occurs predominantly during the larval phase. This reflects the added value of accounting for strong environmental influences on early life stage. However, considering the novelty of our approach, combined with the fact that any statistical correlation cannot ensure causation^81^, we acknowledge that some of the correlations we attempted to detect here might lack a realistic biological causal link. Nevertheless, the derived findings should serve as food for thought for future studies and should definitely be explored further through the application of spatially informed stock assessments. The application of spatially explicit models is highly valuable and a realistic first step would be to compare key assessment metrics (e.g., recruitment, biomass) with and without the inclusion of connectivity^112^. There are five primary generalized stock assessment platforms (such as the stock synthesis^113^ capable of incorporating spatial population structure and connectivity that have been used to provide tactical management advice for national and international Fisheries Management Organizations^18^. Properly parameterizing active and passive movements as well as integrating multidisciplinary approaches are key to advancing the next generation of spatial stock assessments^18,22^.

Sustainable development necessitates informed decisions on where, when, and how to conserve, restore and manage marine species and ecosystems^114^. The need to align management and assessment units with real biological population boundaries has already been highlighted^21^, and is also critical for informing conservation strategies^115^. Understanding the geographical distribution of nurseries and their connectivity to spawning grounds is crucial for determining the spatial scales over which fisheries should be managed and for designing and implementing marine protected area (MPA) networks in the overfished Mediterranean Sea^43^, including Fisheries Restricted Areas^115^(FRAs). Nonetheless, it is essential to address the numerous technical, administrative, and legislative challenges inherent in transitioning to next-generation assessment and management frameworks, particularly in areas like the Mediterranean Sea, where stock boundaries typically -at least in non-European countries- largely correspond to national or subnational boundaries^52^. Such a transition -notably for transnational units requiring international collaboration and standardized data collection frameworks - would necessitate a shift away from nation - or region-specific closed practices and protocols. Encouragingly, certain examples of cross-unit and transnationally managed stocks in the Mediterranean Sea already exist, such as the hake, the deep-water rose shrimp and others^57,115^, but predominantly concern European countries, rather that African and Asian counterparts. However, even these “success stories” require further advancements^57,115^, with the establishment of effective international agreements likely remaining a top priority.

## Conclusion

Understanding species sensitivity to such cumulative and emerging human pressures requires a mechanistic understanding of dispersal processes, such as revealing and monitoring dispersal corridors and source-sink dynamics^8^. This is crucial for developing efficient conservation and management measures^116,117,118^. By revealing and quantifying intra-population connectivity processes, our study not only challenges the existing paradigm of separate disconnected stock units but also underscores the significance of integrating ELS-mediated connectivity into fisheries assessment and providing a sound scientific basis defining the complex spatial structure (i.e., metapopulation) of the species in the region. The results have broader implications for the sustainable management of fisheries resources in the Mediterranean and beyond. By uncovering the high levels of both between- and within-stock connectivity from the highly advected pelagic ELS to the resident adults, this research contributes valuable knowledge that can inform more effective assessment and management strategies, ultimately fostering the resilience of Mediterranean fisheries in the face of ongoing environmental and human perturbations.

## Supplementary Information

Below is the link to the electronic supplementary material.

Supplementary Material 1

## Data Availability

Part of the data is included in the supplementary information files, while the remaining data is available upon reasonable request by contacting georgios.kerametsidis@ieo.csic.es or jm.hidalgo@ieo.csic.es. The daily zero-inflated filtered connectivity matrices that were used to derive the connectivity indices as well as the daily connectivity indices and particle transports for the 10 years have been uploaded to https://doi.org/10.5281/zenodo.15916643.
