# Supplementary material for: Evidence of within- and between-stock connectivity in Mediterranean fisheries challenges the stock unit paradigm
Source: Sci Rep. 2026 Jun 2;16:25169. doi: 10.1038/s41598-026-47826-7 (PMC13470241; doi:10.1038/s41598-026-47826-7)
Supplement: Supplementary file 1 [file 41598_2026_47826_MOESM1_ESM.docx]

**Evidence of within- and between-stock connectivity in Mediterranean fisheries challenges the stock unit paradigm**

Georgios Kerametsidis^1^*, Manuel Hidalgo^1^*, Baptiste Mourre^2,3^, Enrico Ser-Giacomi^4^, Lucía López-López^5^, Antonio Esteban^6^, Encarnación García^6^, Miguel Vivas^6^, Angélique Jadaud^7^, Grégoire Certain^7^, Vincent Rossi^8^

^1^Spanish Institute of Oceanography (IEO, CSIC), Balearic Oceanographic Center, Group of Ecosystem Oceanography (GRECO), Palma, Balearic Islands, Spain

^2^IMEDEA, Mediterranean Institute for Advanced Studies (CSIC-UIB), Esporles, Balearic Islands, Spain

^3^SOCIB, Balearic Islands Coastal Observing and Forecasting System, Palma, Balearic Islands, Spain

^4^IFISC, Institute for Cross-Disciplinary Physics and Complex Systems (CSIC-UIB), Palma, Balearic Islands, Spain

^5^Spanish Institute of Oceanography (IEO, CSIC), Oceanographic Center of Santander, Group of Ecosystem Oceanography (GRECO), Santander, Cantabria, Spain

^6^Spanish Institute of Oceanography (IEO, CSIC), Oceanographic Center of San Pedro del Pinatar, Lo Pagán, Murcia, Spain

^7^MARBEC, Univ Montpellier, CNRS, Ifremer, IRD, Sète, France

^8^Mediterranean Institute of Oceanography, CNRS, Aix Marseille Univ., Univ. Toulon, IRD, Marseille, France

*Corresponding authors: Georgios Kerametsidis (email: georgios.kerametsidis@ieo.csic.es), Manuel Hidalgo (email: jm.hidalgo@ieo.csic.es), Muelle de Poniente, s/n, 07015 Palma, Balearic Islands, tel: +34 971 13 37 20

# SI-1. Time series of Local Retention (LR), Self-Recruitment (SR) and Source-Sink Strength (SSS)

**Figure S1**. Annual variation of the three main connectivity indices for the 4 subareas: (a) Local Retention (LR), (b) Self-recruitment (SR), (c) Source-sink strength (SSS).

# SI-2. High spawning quality areas

**Figure S2**. Representation of high spawning quality areas. Only areas where the spawning quality exceeds the first quartile (>0.25) are portrayed. For further details, the reader is referred to Fig. 4 from Kerametsidis et al.^1^. The figure was created in R (Version 4.2.2, https://www.R-project.org).

# SI-3. Time series of Transports

**Figure S3**. Interannual variation of successful imports (total number of particles arriving in a favourable for settlement locality) per subarea. Subareas are indicated as follows: GoL – Gulf of Lion (a), CC – Catalan Coast (b), GoV – Gulf of Valencia (c), and GoA – Gulf of Alicante (d).

**Figure S4**. Interannual variation of successful exports (total number of particles spawned and departing from a locality and ending up in a favourable area for settlement) per subarea. Subareas are indicated as follows: GoL – Gulf of Lion (a), CC – Catalan Coast (b), GoV – Gulf of Valencia (c), and GoA – Gulf of Alicante (d).

# SI-4. Sensitivity analysis of the transport indices to the applied filters

To explore the impact of the applied filters (i.e. “spawning quality filter”, “settlement suitability filter” and “temporal filter”) on the ELS connectivity, we tested three scenarios where we removed one or several of the three filters that we applied in our main analyses. First, in the first scenario (*B200_Tf_Nf*), we replaced the “spawning quality filter” based on from Kerametsidis et al.^1^ with a simple bathymetric filter from the literature based on the assumption that all sites with a bathymetry equal or lower than 200m are suitable spawning sites while maintaining the “temporal filter”. In the second scenario (*Sf_Nf*), we removed the “temporal filter”, assuming that each release date from the 1^st^ of May to the 31^st^ of July are equally likely to produce the same number of particles (i.e. no spawning peak on the 1^st^ of June), while maintaining the “spawning quality filter”. Finally, in the third scenario (*B200_Nf*), we replaced the “spawning quality filter” with the simple 200m bathymetric filter (similar to the *B200_tf_Nf* scenario) and we also removed the “temporal filter”. In all scenarios, the “settlement suitability filter” *Nf* was retained.

The application of the filters yielded the highest transport from the GoL to the CC, which in the alternative, less biologically informed scenarios would be decreased by 3 to 16% (Fig S5). The removal of the spawning quality filter would result in major transport of particles from the CC to the GoV (Fig. S5). Notably, in the alternative 3 scenarios, the GoA appears to be more connected to the GoV through a higher unidirectional transport of particles.

Regarding the 3 main connectivity indices, they seem to be overall unaffected in the 3 alternative less biologically informed scenarios with the only notable difference observed when the “Temporal filter” is removed (i.e. in *Sf_Nf* and *B200_Nf* scenarios) and the *SR* in the GoL is increased while the *SR* in the GoA is decreased (Fig. S6).

For each year from 2009 to 2018, the daily zero-inflated connectivity matrices indicating the inter-node particle transports as well as the daily connectivity indices (see below) have been reported online at https://doi.org/10.5281/zenodo.15916643, for each of the three sensitivity scenarios described above.

**Figure S5**. Mean transports of exports (arrows) and imports (pies). Percentages are based on the average total number of particles either departing from (arrows) or arriving into (pies) each subarea. Arrows without labels represent outgoing transport flows greater than 3% but less than or equal to 10%. Unlabelled segments in the pie charts represent incoming transport flows of 10% or less. Subareas are indicated as follows: GoL – Gulf of Lion, CC – Catalan Coast, GoV – Gulf of Valencia, and GoA – Gulf of Alicante. (a) All filters applied, (b) Spawning quality filter replaced, (c) Temporal filter removed, (d) Spawning filter replaced and temporal filter removed. The figure was created in R (Version 4.2.2, https://www.R-project.org).

**Figure S6**. Average values of the three connectivity indices: Local Retention (LR), Self-Recruitment (SR), Source-Sink Strength (SSS) in a combined plot. Subareas are indicated as follows: GoL – Gulf of Lion, CC – Catalan Coast, GoV – Gulf of Valencia, and GoA – Gulf of Alicante. (a) All filters applied, (b) Spawning quality filter replaced, (c) Temporal filter removed, (d) Spawning quality filter replaced and temporal filter removed.

# SI-5. Spearman’s rank correlation coefficients and linear regression models

Successive Spearman’s rank correlations coefficient tests (*r*) were performed between each of the “Demographic variables” and all the “Connectivity variables”, for the GSA06 and the GSA07. Significant correlations (|*r|*>0.4, p<0.05) were detected for Spawning Stock Biomass (*S*) and the residuals of Ricker stock-recruitment relationship for 1- and 2-yr lag for the GSA06 and for Recruitment Success (*RS*) (1-yr lag) and the residuals of Ricker stock-recruitment relationship for GSA07 (1- and 2-yr lag) (Fig. S7-8). No significant correlations were detected for the rest of the “Demographic variables”. The Spearman’s method was selected because many of the variables were not normally distributed.

**Figure S7**. Spearman's rank correlation coefficients (r) for the GSA06 between: (a) the Connectivity variables in 1-yr lag and the Spawning Stock Biomass (S), (b) the Connectivity variables in 2-yr lag and the Spawning Stock Biomass (S), (c) the Connectivity variables in 1-yr lag and the residuals of Ricker’s Stock-Recruitment (S-R) relationship, and (d) the Connectivity variables in 2-yr lag and the residuals of Ricker’s Stock-Recruitment (S-R) relationship. OUTWvec (exports): total of departing particles; INWvec (imports): total of incoming particles; T: Transport strength (number of transported particles); LR: Local Retention; SR: Self Recruitment; GSA07: Geographic Subarea 7 (Gulf of Lion); GSA06: Geographic Subarea 6 (Northern Mediterranean Spanish Coast); GoL – Gulf of Lion; CC – Catalan Coast; GoV – Gulf of Valencia; GoA – Gulf of Alicante. Demography and connectivity indices on the y-axes are calculated over the entire larval dispersal period (May–July), unless otherwise specified, to emphasize peak spawning months (May and/or June).

**Figure S8**. Spearman's rank correlation coefficients (r) for the GSA07 between: (a) the Connectivity variables in 1-yr lag and the Recruitment Success (RS), (b) the Connectivity variables in 1-yr lag and the residuals of Ricker’s Stock-Recruitment (S-R) relationship, and (d) the Connectivity variables in 2-yr lag and the residuals of Ricker’s Stock-Recruitment (S-R) relationship. OUTWvec (exports): total of departing particles; INWvec (imports): total of incoming particles; T: Transport strength (number of transported particles); LR: Local Retention; SR: Self Recruitment; GSA07: Geographic Subarea 7 (Gulf of Lion) ; GSA06: Geographic Subarea 6 (Northern Mediterranean Spanish Coast); GSA05: Geographic Subarea 5 (Balearic Islands); GoL – Gulf of Lion; CC – Catalan Coast; GoV – Gulf of Valencia; GoA – Gulf of Alicante. Demography and connectivity indices on the y-axes are calculated over the entire larval dispersal period (May–July), unless otherwise specified, to emphasize peak spawning months (May and/or June).

Given the short length of the time series (2009-2018), the number of applicable statistical approaches was limited, and our primary objective was to explore whether larval dispersal metrics could be directly linked to outputs from stock assessment. In addition to Spearman’s rank correlation test (which is characterized by minimal distributional assumptions), we also applied simple linear regression models to assess statistical relationships between demographic variables and connectivity variables and examine whether the obtained statistical links differ or not with the ones obtained from correlation tests. All demographic variables were normally distributed, with the exception of spawning stock biomass (SSB) with a one-year lag, which was excluded from the linear regression analysis.

Owing to the limited number of observations, we restricted the analysis to models including a single explanatory variable. In total, we identified twelve statistically significant relationships (four for GSA06 and eight for GSA07), fewer than those detected using Spearman’s correlation. Of these twelve linear models, eight supported the same associations identified by the correlation tests (Table S1). Notably, the linear regression analysis also revealed a relationship involving RS in GSA06, which was not detected by the Spearman correlation analysis (Table S1).

Kerametsidis et al. 2026 (in prep) builds upon this present study and directly incorporates connectivity dynamics into a spatially explicit simulation framework to explore the effects and (bias generated) by accounting or not for early life dispersal in stock assessment models.

**Table S1**. Covariates of the best Linear Regression Models obtained to model Recruitment (R), Recruitment Success (RS), the residuals from Stock-Recruitment Relationship (S_R_resid) and Spawning Stock Biomass (S). Adjusted R2 (R^2^_adj_), AICc and ΔAICc (representing the difference in AIC between a given model and the best model for each group of models) for each model are also displayed. AICc: corrected Akaike Information Criterion; OUTWvec (exports): total of departing particles; INWvec (imports): total of incoming particles; T: Transport strength (number of transported particles); LR: Local Retention; SR: Self Recruitment; GSA07: Geographic Subarea 7 (Gulf of Lion) ; GSA06: Geographic Subarea 6 (Northern Mediterranean Spanish Coast); GSA05: Geographic Subarea 5 (Balearic Islands); GoL – Gulf of Lion; CC – Catalan Coast; GoV – Gulf of Valencia; GoA – Gulf of Alicante. Demography and connectivity indices are calculated over the entire larval dispersal period (May–July), unless otherwise specified, to emphasize peak spawning months (May and/or June). Red colour indicates statistical links identified exclusively by linear models for a given response variable, for which no significant associations were detected using Spearman’s correlation tests; blue colour indicates links that were also identified using Spearman’s correlation analysis.

| **Response variable** | **Explanatory variable(s)** | **R^2^_adj_** | **AICc** | **ΔAICc** |
| --- | --- | --- | --- | --- |
| **RS_GSA06_lag1** | **SR_GoV** | **0.40** | **87.48** | **0.00** |
| **RS_GSA07_lag1** | SR_June_CC | 0.39 | 99.67 | 0.00 |
|  | **T_CC_to_GoV_May** | **0.35** | **100.42** | **0.75** |
| **S_R_resid_GSA06_lag1** | **T_CC_to_GoV_May** | **0.35** | **228.86** | **0.00** |
| **S_R_resid_GSA06_lag2** | **INWvec_GSA07_June** | **0.61** | **221.69** | **0.00** |
| **S_R_resid_GSA07_lag1** | **INWvec_GSA06_May_June** | **0.65** | **207.50** | **0.00** |
|  | **LR_GSA07_May_June** | **0.62** | **208.22** | **0.72** |
|  | **OUTWvec_GSA07_May_June** | **0.61** | **208.44** | **0.94** |
|  | SR_GSA06_May_June | 0.61 | 208.48 | 0.98 |
|  | **SR_June_CC** | **0.60** | **208.83** | **1.33** |
|  | **T_GoL_to_CC_May_June** | **0.59** | **208.87** | **1.38** |
| **S_GSA06_lag2** | T_GoV_to_GoA_May | 0.63 | 108.11 | 0.00 |

# SI-6. Sensitivity analysis of the depth layer selection

Red mullet larvae are typically found in the surface layer (i.e. 0-10m) but, occasionally during the night, they can be found in deeper waters, up to 20m ^2^. Although horizontal current speeds are of several orders of magnitude higher than vertical current speeds^3^, we simulated ELS dispersal in a higher depth (=30m) in 2013 and compared the outcomes with the *default* simulation depth (=10m) to explore any potential effect on the transport of particles. We calculated the daily successful imports (total number of particles arriving in a locality) (Fig. S9) and exports (total number of particles spawned and departing from a locality) (Fig. S10) per subarea after all filters were applied and calculated the correlation coefficients between the *default* and the *high-depth* scenarios. Finally, we visualized the transport ratios between biologically relevant pairs of subareas for both scenarios (Fig. S11).

**Figure S9**. Successful imports (total number of particles arriving in a favourable for settlement locality) for the default (grey) and high-depth (black) scenarios. Dotted lines represent the daily imports while solid lines represent the monthly averages. Subareas are indicated as follows: GoL – Gulf of Lion, CC – Catalan Coast, GoV – Gulf of Valencia, and GoA – Gulf of Alicante.

**Figure S10**. Successful exports (total number of particles spawned and departing from a locality and ending up in a favourable area for settlement) for the default (grey) and high-depth (black) scenarios. Dotted lines represent the daily imports while solid lines represent the monthly averages. Subareas are indicated as follows: GoL – Gulf of Lion, CC – Catalan Coast, GoV – Gulf of Valencia, and GoA – Gulf of Alicante.

All correlation coefficients were high to very high in the successful imports and exports between the *default* and *high-depth* scenarios, with the exception the successful imports in the GoA (Fig. S8). Overall, higher imports and exports were observed under the *default* scenario.

In regard to the transport ratios of particles, similar values were observed under both scenarios with the only notable exceptions (difference higher than 10%), detected only in the transport from the GoL to the CC in May and from the GoA to GoV in June (Fig. S10), where higher values were observed under the *default* scenario.

Overall, our sensitivity test reveals strong similarities in successful imports and exports, as well as in transport ratios, supporting the hypothesis that minor variations in the selected dispersal depth have minimal impact on the outcomes of connectivity studies.

**Figure S11**. Transport ratios of particles for the *default* (grey) and *high-depth* (black) scenarios. Points represent the monthly transport ratios while solid lines represent the annual averages. Subareas are indicated as follows: GoL – Gulf of Lion, CC – Catalan Coast, GoV – Gulf of Valencia, and GoA – Gulf of Alicante.

# SI*-*Table S2. Summary of the white muscle tissue samples

| **ID** | **Subarea** | **GSA** | ***δ*^13^C** | ***δ*^15^Ν** | **Lat** | **Lon** | | **Mass (g)** | **Length (cm)** |
| --- | --- | --- | --- | --- | --- | --- | --- | --- | --- |
| Castellon.8 | GoV | 6 | -19.18 | 12.00 | 39.96 | 0.13 | 50.14 | | 16.1 |
| GLyo.1 | GoL | 7 | -17.16 | 9.97 | 42.68 | 3.58 | 72.5 | | 19 |
| GLyo.11 | GoL | 7 | -17.03 | 10.94 | 42.68 | 3.58 | 47.5 | | 16.9 |
| GLyo.12 | GoL | 7 | -18.42 | 10.56 | 42.68 | 3.58 | 37 | | 15.6 |
| GLyo.13 | GoL | 7 | -18.66 | 11.12 | 42.68 | 3.58 | 33.5 | | 15.5 |
| GLyo.14 | GoL | 7 | -19.69 | 10.52 | 42.68 | 3.58 | 23 | | 13.7 |
| GLyo.15 | GoL | 7 | -18.84 | 10.76 | 42.68 | 3.58 | 41.5 | | 16 |
| GLyo.2 | GoL | 7 | -18.03 | 11.22 | 42.68 | 3.58 | 139 | | 23.5 |
| GLyo.20 | GoL | 7 | -18.99 | 10.27 | 42.68 | 3.58 | 32.5 | | 15.5 |
| GLyo.29 | GoL | 7 | -19.69 | 10.51 | 42.68 | 3.58 | 21.5 | | 13.3 |
| GLyo.3 | GoL | 7 | -18.88 | 10.66 | 42.68 | 3.58 | 50.5 | | 17.2 |
| GLyo.31 | GoL | 7 | -19.06 | 10.53 | 42.68 | 3.58 | 32 | | 15.1 |
| GLyo.34 | GoL | 7 | -19.68 | 10.74 | 42.68 | 3.58 | 25.5 | | 14 |
| GLyo.4 | GoL | 7 | -18.79 | 10.62 | 42.68 | 3.58 | 59.5 | | 18.3 |
| GLyo.6 | GoL | 7 | -18.18 | 10.55 | 42.68 | 3.58 | 95 | | 21.2 |
| GLyo.7 | GoL | 7 | -18.62 | 9.97 | 42.68 | 3.58 | 53 | | 17.5 |
| H123.1 | GoV | 6 | -17.29 | 12.51 | 39.88 | 0.47 | 105.45 | | 20.7 |
| H123.2 | GoV | 6 | -19.99 | 11.32 | 39.88 | 0.47 | 85.25 | | 19.2 |
| H123.5 | GoV | 6 | -19.80 | 11.21 | 39.88 | 0.47 | 59.69 | | 17.4 |
| H123.6 | GoV | 6 | -17.87 | 12.09 | 39.88 | 0.47 | 103.33 | | 20.5 |
| H123.7 | GoV | 6 | -18.37 | 11.82 | 39.88 | 0.47 | 101.92 | | 20.5 |
| H124.1 | GoV | 6 | -18.46 | 12.52 | 40.02 | 0.56 | 113.98 | | 20.3 |
| H124.2 | GoV | 6 | -18.16 | 12.53 | 40.02 | 0.56 | 65.02 | | 16.7 |
| H124.3 | GoV | 6 | -50.81 | 12.45 | 40.02 | 0.56 | 71.84 | | 17.8 |
| H124.4 | GoV | 6 | -32.88 | -1.73 | 40.02 | 0.56 | 115.13 | | 20.6 |
| H126.15 | GoV | 6 | -19.08 | 11.60 | 39.83 | 0.70 | 91.51 | | 20 |
| H126.2 | GoV | 6 | -17.78 | 11.54 | 39.83 | 0.70 | 74.33 | | 18.6 |
| H126.24 | GoV | 6 | -18.24 | 12.01 | 39.83 | 0.70 | 90.2 | | 20.5 |
| H126.5 | GoV | 6 | -18.47 | 11.21 | 39.83 | 0.70 | 81.24 | | 19.3 |
| H126.9 | GoV | 6 | -18.62 | 12.12 | 39.83 | 0.70 | 58.49 | | 17 |
| H147.1 | CC | 6 | -18.31 | 11.16 | 41.67 | 3.36 | 133.81 | | 22 |
| H147.2 | CC | 6 | -18.47 | 12.10 | 41.67 | 3.36 | 121.03 | | 21.7 |
| H148.1 | CC | 6 | -18.52 | 10.82 | 41.73 | 3.25 | 47.51 | | 16.6 |
| H148.2 | CC | 6 | -20.39 | 10.21 | 41.73 | 3.25 | 53.96 | | 16.8 |
| H148.3 | CC | 6 | -19.79 | 10.76 | 41.73 | 3.25 | 52.4 | | 17.3 |
| H148.4 | CC | 6 | -18.72 | 10.83 | 41.73 | 3.25 | 123.31 | | 22.2 |
| H148.5 | CC | 6 | -17.80 | 10.89 | 41.73 | 3.25 | 125.68 | | 21.7 |
| H148.6 | CC | 6 | -18.53 | 11.30 | 41.73 | 3.25 | 68.72 | | 18.5 |
| H149.1 | CC | 6 | -19.25 | 10.70 | 41.81 | 3.15 | 51.67 | | 17.4 |
| H149.10 | CC | 6 | -18.90 | 10.37 | 41.81 | 3.15 | 71.32 | | 18.4 |
| H149.11 | CC | 6 | -18.01 | 10.87 | 41.81 | 3.15 | 56.79 | | 17.7 |
| H149.18 | CC | 6 | -18.62 | 11.02 | 41.81 | 3.15 | 66.96 | | 17.8 |
| H149.21 | CC | 6 | -19.32 | 10.87 | 41.81 | 3.15 | 50 | | 16.9 |
| H149.8 | CC | 6 | -16.98 | 11.64 | 41.81 | 3.15 | 121.38 | | 21.8 |
| H149.9 | CC | 6 | -18.05 | 10.84 | 41.81 | 3.15 | 57.42 | | 16.9 |
| H86.1 | GoA | 6 | -20.87 | 11.96 | 38.02 | -0.33 | 27.56 | | 20.4 |
| H86.11 | GoA | 6 | -18.65 | 11.72 | 38.02 | -0.33 | 45.09 | | 16.9 |
| H86.13 | GoA | 6 | -18.54 | 12.12 | 38.02 | -0.33 | 98.09 | | 20.7 |
| H86.3 | GoA | 6 | -19.03 | 11.73 | 38.02 | -0.33 | 56.52 | | 17.5 |
| H86.5 | GoA | 6 | -19.47 | 12.16 | 38.02 | -0.33 | 51.23 | | 16.8 |
| H86.7 | GoA | 6 | -18.96 | 11.95 | 38.02 | -0.33 | 67.21 | | 18.4 |
| H86.9 | GoA | 6 | -17.79 | 11.86 | 38.02 | -0.33 | 121.88 | | 21.6 |
| H87.11 | GoA | 6 | -18.41 | 12.28 | 38.09 | -0.24 | 49.33 | | 17.2 |
| H87.14 | GoA | 6 | -17.89 | 11.64 | 38.09 | -0.24 | 87.13 | | 19.9 |
| H87.17 | GoA | 6 | -19.11 | 10.61 | 38.09 | -0.24 | 72.02 | | 18.2 |
| H87.19 | GoA | 6 | -17.39 | 11.97 | 38.09 | -0.24 | 112.22 | | 22 |
| H87.21 | GoA | 6 | -18.12 | 12.03 | 38.09 | -0.24 | 84.29 | | 20.3 |
| H87.23 | GoA | 6 | -17.98 | 12.05 | 38.09 | -0.24 | 139.08 | | 22.8 |
| H87.25 | GoA | 6 | -17.46 | 11.97 | 38.09 | -0.24 | 86.14 | | 18.9 |
| H87.8 | GoA | 6 | -17.95 | 11.56 | 38.09 | -0.24 | 74.05 | | 18.5 |

GSA: Geographic Subarea; GoV: Gulf of Valencia; GoL: Gulf of Lion; CC: Catalan Coast; GoA: Gulf of Alicante; *δ*^13^C: Carbon-13 stable isotope; *δ*^15^N: Nitrogen-15 stable isotope

# References

1.	Kerametsidis, G. *et al.* Cross-scale environmental impacts across persistent and dynamic aggregations within a complex population: implications for fisheries management. *Canadian Journal of Fisheries and Aquatic Sciences* **81**, 268–284 (2024).

2.	Sabatés, A., Zaragoza, N. & Raya, V. Distribution and feeding dynamics of larval red mullet (Mullus barbatus) in the NW Mediterranean: The important role of cladocera. *J Plankton Res* **37**, 820–833 (2015).

3.	d’Ovidio, F., Fernández, V., Hernández-García, E. & López, C. Mixing structures in the Mediterranean Sea from finite-size Lyapunov exponents. *Geophys Res Lett* **31**, (2004).
